# Structure‐specific Mini‐prion Model for Alzheimer's Disease Tau Fibrils

**DOI:** 10.1002/alz70861_108374

**Published:** 2025-12-23

**Authors:** Vishnu Vijayan, Gregory Merz, Athena Quddus, Andrew P Longhini, Michael Vigers, Kristi Lynn S Nakagawa, Kenneth S. Kosik, Daniel Southworth, Songi Han

**Affiliations:** ^1^ University of California Santa Barbara, Santa Barbara, CA USA; ^2^ University of California San Francisco, San Francisco, CA USA; ^3^ Northwestern University, Evanston, IL USA

## Abstract

**Background:**

One of the most significant discoveries of the past decade is that tau protein assembles into amyloid fibrils with distinct, disease‐specific hallmark structures in each tauopathy. Tau pathology is thought to progress via a "prion‐like" mechanism, where pathological tau seeds recruit naive tau to form aggregates. Developing synthetic tauopathy fibril models with prion properties as targets for diagnostic and therapeutic strategies remains a major challenge due to the lack of rational methods to generate disease‐specific fibril folds and limited structural biology tools to screen heterogeneous fibril populations.

**Method:**

We employed a novel approach using mini‐prions to generate seeded tau fibrils, leveraging the hypothesis that fibril seeding initiates from a localized critical motif within the seed structure. The mini‐prion peptides have a limited number of amino acids that explore a significantly reduced conformational landscape, facilitating rational manipulation toward adopting a thermodynamically stable, homogeneous seed structure, which is challenging to achieve with larger tau. Double Electron‐Electron Resonance (DEER) spectroscopy was employed to characterize the evolving populations of heterogeneous structures formed during the seeded assembly.

**Result:**

A 16‐amino‐acid mini‐AD prion peptide was designed and assembled into a critical fibril core motif as found in Alzheimer’s Disease (AD) tau fibrils. These fibrils serve as seeds with exquisite prion competency, both in vitro and in tau biosensor cells, capable of recruiting tau constructs up to ten times larger to generate disease‐relevant fibrils. By manipulating salt conditions in the reaction‐buffer (MgCl_2_ versus NaCl), distinct tau fibril folds of AD and Chronic Traumatic Encephalopathy (CTE) were generated through mini‐AD seeding as confirmed through morphological analyses and structural studies using DEER spectroscopy. Multigenerational seeding with the seeded fibrils amplified the quantity of AD‐like paired helical filaments. DEER studies confirmed the preservation of the AD‐like structure across generations, demonstrating the seeding and templating competency of these fibrils and shedding light on the mechanisms of disease progression in human brains.

**Conclusion:**

The mini‐AD seeds and the seeded fibrils, with their remarkable templating capabilities, offer a novel strategy to design model tau fibril prions to achieve and investigate the seeded propagation of tau in vitro, in cellular and animal models.

